# Trends in female participation at the Brazilian Congress of Ophthalmology, 2016–2023

**DOI:** 10.5935/0004-2749.2024-0333

**Published:** 2025-09-10

**Authors:** Stéphanie Pessoa Regueira, Taíse Maria Clemente de Araújo, Julia Melo Silva Santiago, Gustavo Rosa Gameiro, Wilma Lelis Barboza, Camila V. Ventura

**Affiliations:** 1 Department of Ophthalmology, Fundação Altino Ventura, Recife, PE, Brazil; 2 Universidade Pernambucana de Saúde, PE, Brazil; 3 Department of Ophthalmology, Bascom Palmer Eye Institute, University of Miami, Miami, FL, USA; 4 Department of Ophthalmology, Escola Paulista de Medicina, Universidade Federal de São Paulo, São Paulo, SP, Brazil; 5 Department of Ophthalmology, Faculdade de Medicina, University de São Paulo, São Paulo, SP, Brazil; 6 Department of Ophthalmology, Faculdade de Medicina, Universidade de Taubaté, Taubaté, SP, Brazil; 7 Department of Ophthalmology, Hospital de Olhos de Pernambuco, Recife, PE, Brazil

**Keywords:** Ophthalmology, Gender equity, Ophthalmologists/statistics & numerical data, Physicians, women/statistics & nu merical data, Leadership, Congresses as topic/statistics & numerical data

## Abstract

**Purpose:**

To assess female participation in the Brazilian Congress of Ophthalmology.

**Methods:**

This retrospective, descriptive-analytical study examined the profiles of individuals involved in the scientific program of the Brazilian Congress of Ophthalmology from 2016 to 2023. Data were provided by the Brazilian Council of Ophthalmology and were categorized by ophthalmology subspecialty, participant role, and geographic region of origin within Brazil. Roles were grouped into three main categories: coordinator, speaker, and moderator/discussant.

**Results:**

Female participation at the congress increased from 33% in 2016 to 42% in 2023, showing an annual upward trend of 1.33 (p<0.001). Around 64% of female participants were from the Southeast region, while 16% were from the Northeast. The coordinator role showed the largest increase in female participation, rising from 22% in 2016 to 40% in 2023 (Slope: 2; p<0.001), followed by the speaker role, which increased from 34% to 44% (Slope: 1.5; p<0.001), and the moderator/discussant role, which rose from 32% to 38% (Slope: 1.24; p=0.0586). Changes in female representation across ophthalmology subspecialties were not statistically significant.

**Conclusion:**

From 2016–2023, female participation in the Brazilian Congress of Ophthalmology increased across most subspecialties and conference roles. Although gender disparity has narrowed, continuous efforts are needed to achieve greater gender equity and equality in ophthalmology conferences.

## INTRODUCTION

Over recent decades, the feminization of the medical profession has become increasingly apparent. In Brazil, the proportion of female physicians rose from 22.3% in 1910 to 46.6% in 2020^(1)^. While this trend reflects a significant rise in the number of women entering medicine, it does not necessarily correspond to a proportional increase in their presence within medical specialties or leadership positions^(2)^.

Ophthalmology is notable for being the third most represented surgical specialty among women^(2)^. In the 1970s, only 4% of ophthalmologists worldwide were women, whereas by 2021, that figure had grown to between 25% and 30%^(3)^. Despite this substantial growth, there is still considerable potential for further advancement^(3)^.

In the Brazilian context, women currently make up 39.9% of practicing ophthalmologists^(1)^. One possible way to assess female representation in leadership and academic roles is to analyze their involvement in professional conferences and congresses^(3)^. A 2022 study examining female participation at the American Academy of Ophthalmology (AAO) annual meeting found a marked increase in female involvement across all subspecialties and conference roles between 2018 and 2021, although the figures remained below anticipated levels^(3)^. Global data show that disparities are more pronounced in certain ophthalmic surgical subspecialties, such as retina and vitreous, while areas like pediatric ophthalmology have traditionally seen higher female participation^(4)^.

Given this context, present study aimed to assess the representation of women in Brazilian ophthalmology by analyzing their participation and roles within the scien tific program of the Brazilian Congress of Ophthalmo logy (CBO) from 2016 to 2023.

## METHODS

This study is retrospective and descriptive-analytical in nature, utilizing secondary data. It analyzed the profiles of participants at the CBO from 2016 to 2023. The CBO is among the principal general ophthalmology congresses in Brazil, drawing attendees from all subspecialties and geographic regions of the country. Consequently, it serves as a representative sample of Brazilian ophthalmologists’ participation within the scientific community. The data used in this analysis were obtained from the CBO via the CBO database covering the years 2016 through 2023.

The demographic information analyzed was self-reported by participants during registration for the CBO annual meetings and included the Brazilian region of origin and binary gender designation (male or female). Individuals who did not respond to the gender question were excluded from the study. Other gender identities, such as trans women, could not be assessed, as these options were not available during the registration process. Participants also self-identified their ophthalmo logy subspecialty at the time of registration.

The data were examined according to the participant’s subspecialty and their role at the congress. The CBO includes a variety of sessions and activities, which may differ in terminology from year to year. To ensure consistency in the analysis, participant roles within the scientific program were categorized into three primary groups: speaker, coordinator, and moderator/discussant.

If an individual participated in multiple activities during the congress, all instances were recorded; however, for the purpose of analyzing participant profiles, each person was counted only once.

### Statistical analysis

Relative frequency distributions were used to analyze trends in female participation over the study period and to compare these trends with the growth in the number of registered ophthalmologists in Brazil during the same timeframe, based on data from the CBO. Simple linear regression models were applied to evaluate changes in female participation across the years and to determine whether these changes reflected a nonzero slope, enabling a more detailed evaluation of the observed trend. Female participation was also analyzed by geographic region, considering proportions within Brazil’s five macroregions. To evaluate changes in participation rates, year-to-year differences in the observed proportions were examined, and the chi-squared test was used to compare proportions. Statistical significance was defined as a p-value less than 0.05. The analyses were performed using Jamovi version 2.3.18, RStudio version 4.3.3, and QGIS version Buenos Aires 3.26.

## RESULTS

**Figure 1:**
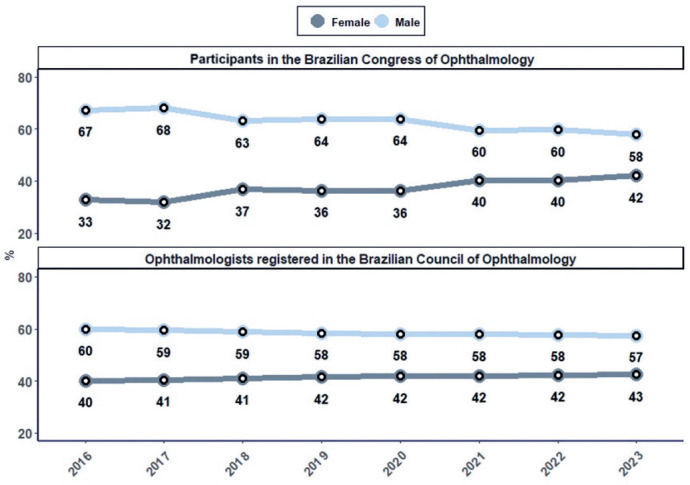
Female participation at the Brazilian Congress of Ophthalmology from 2016 to 2023, compared with the percentage of female ophthalmologists practicing in Brazil during the same period.

In 2016, women accounted for 40% of the ophthalmologists registered with the CBO and made up 33% (n=218) of the participants in the CBO scientific program. By 2023, the percentage of female ophthalmologists had increased to 43% (n=311), accompanied by a rise in female representation in the scientific program to 42% was noted (Figure 1).

The average number of participations in the scientific program roles (speaker, moderator/discussant, coordinator) per female participant was 2.4 ± 2.3 (range, 1–18) in 2016 and 2.2 ± 1.6 (range, 1–9) in 2023. For male participants, the averages were 2.8 ± 2.7 (range, 1–25) in 2016 and 2.4 ± 1.8 (range, 1–12) in 2023, with p-values of 0.798 and 0.988, respectively.

**Figure 2:**
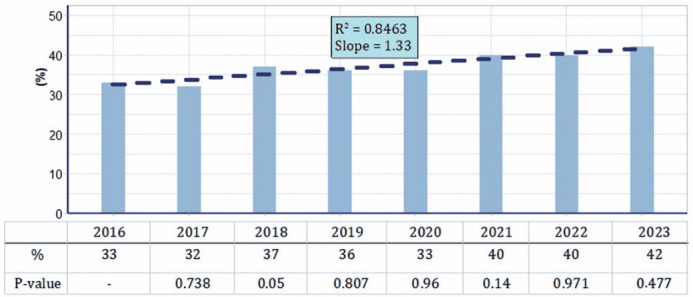
Trend chart showing female participation at the Brazilian Congress of Ophthalmology from 2016 to 2023.

**Figure 3:**
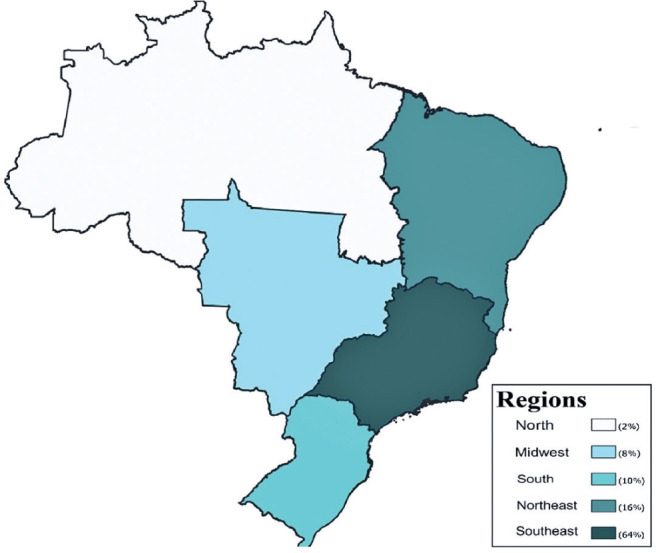
Regional distribution of female participants by their area of origin from 2016 to 2023.

From 2016 to 2023, the annual growth rate of female participation in the CBO was 1.33% (p<0.001) (Figure 2). During this period, approximately 64% of the female ophthalmologists participating in the scientific program were from the Southeast region, followed by 16% from the Northeast region (Figure 3).

**Figure 4:**
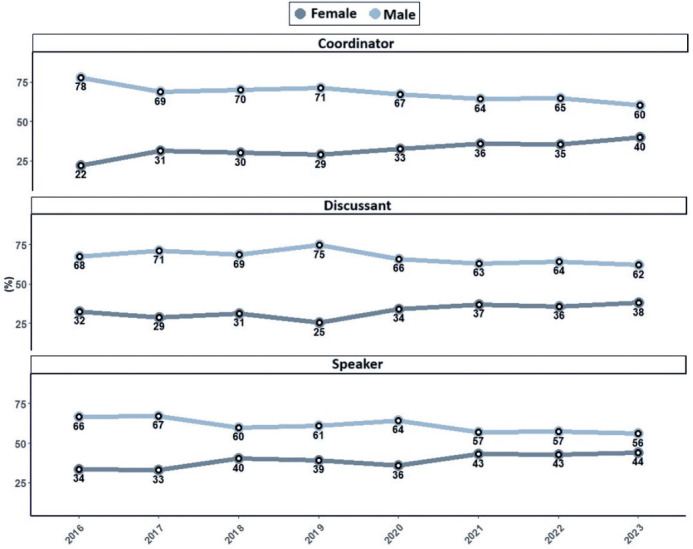
Percentage of male and female participants in the roles of coordinator, moderator, and speaker at the Brazilian Congress of Ophthalmology between 2016 and 2023.

**Figure 5:**
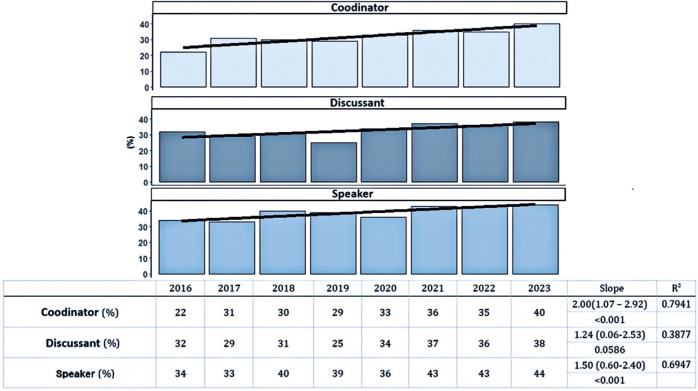
Percentage and trend analysis of female participation in coordinator, moderator, and speaker roles at the Brazilian Congress of Ophthalmology from 2016 to 2023.

The analysis of female participation growth across different conference roles (speaker, moderator/discussant, and coordinator) showed a statistically significant increase in the coordinator and speaker categories. Among these, the coordinator role exhibited the most notable growth. In 2016, women represented 22% of coordinators, increasing to 40% by 2023 (Figure 4). This change corresponds to a significant upward trend over time (Slope: 2.00; p<0.001). The proportion of female speakers rose from 34% in 2016 to 44% in 2023, with a trend analysis indicating an annual growth rate of 1.50% (p<0.001, indicating a slope significantly different from zero). The moderator/discussant role showed the smallest increase, rising from 32% female participation in 2016 to 38% in 2023. The corresponding annual growth rate was 1.24% (p=0.0586), which did not reach statistical significance (Figure 5).

No statistically significant differences were observed in female participation across the various ophthalmology subspecialties during the study period (Table 1).

**Table 1:** Percentage of female participants in the Brazilian Congress of Ophthalmology by specialty from 2016 to 2023 and the proportion of female participants relative to the total number of attendees during the same period

Subspecialty	Proportion of female participation n/total (%)							
	2016	2017	2018	2019	2020	2021	2022	2023
Cataract	13/83 (15.7)	17/96 (17.7)	15/95 (15.8)	25/165 (15.2)	11/60 (18.3)	5/51 (9.8)	11/73 (15.1)	16/82 (19.5)
Cornea	28/77 (36.4)	27/72 (37.5)	28/78 (35.9)	44/112 (39.3)	20/62 (32.3)	22/63 (34.9)	31/84 (36.9)	37/77 (48.1)
Strabismus + Pediatric ophthalmology	41/62 (66.1)	55/150 (36.7)	49/72 (68.1)	42/62 (67.7)	27/42 (64.3)	27/40 (67.5)	62/84 (73.8)	46/63 (73.0)
Refractive surgery	18/77 (23.4)	15/71 (21.1)	14/61 (23.0)	19/62 (30.6)	15/42 (35.7)	15/38 (39.5)	16/53 (30.2)	22/56 (39.3)
Uveitis	13/37 (35.1)	16/42 (38.1)	17/36 (47.2)	16/60 (26.7)	12/22 (54.5)	14/29 (48.3)	18/37 (48.6)	20/46 (43.5)
Neuro-ophthalmology	8/25 (32.0)	8/22 (36.4)	10/28 (35.7)	11/29 (37.9)	9/23 (39.1)	6/20 (30.0)	16/40 (40.0)	10/30 (33.3)
Oculoplastic	25/57 (43.9)	30/63 (47.6)	37/80 (46.2)	38/77 (49.4)	26/56 (46.4)	25/43 (58.1)	40/72 (55.6)	38/66 (57.6)
Oncology	11/22 (50.0)	10/26 (38.5)	12/25 (48.0)	8/25 (32.0)	7/14 (50.0)	13/27 (48.1)	13/29 (44.8)	14/33 (42.4)
Retina	16/105 (15.2)	16/91 (17.6)	8/72 (11.1)	24/126 (19.0)	12/76 (15.8)	15/65 (23.1)	19/81 (23.5)	19/84 (22.6)
Glaucoma	15/78 (19.2)	16/79 (20.3)	24/98 (24.5)	28/103 (27.2)	23/91 (25.3)	21/72 (29.2)	20/88 (22.7)	22/81 (27.2)

Subspecialty	Slope (p-value of nonzero slope)							
	2016–2017	2017–2018	2018–2019	2019–2020	2020–2021	2021–2022	2022–2023	2016–2023
Cataract	2.0 (0.86)	-1.9 (0.87)	-0.6 (1.00)	3.1 (0.71)	-8.5 (0.31)	5.3 (0.55)	4.4 (0.60)	3.8 (0.66)
Cornea	1.1 (1.00)	-1.6 (0.97)	3.4 (0.74)	-7.0 (0.44)	2.6 (0.90)	2.0 (0.94)	11.2 (0.20)	11.7 (0.20)
Strabismus + Pediatric ophthalmology	-29.4 (0.00)	31.4 (0.00)	-0.4 (1.00)	-3.4 (0.87)	3.2 (0.94)	6.3 (0.60)	-0.8 (1.00)	6.9 (0.52)
Refractive surgery	-2.3 (0.89)	1.9 (0.96)	7.6 (0.44)	5.1 (0.74)	3.8 (0.91)	-9.3 (0.48)	9.1 (0.42)	15.9 (0.07)
Uveitis	3.0 (0.96)	9.1 (0.56)	-20.5 (0.06)	27.8 (0.03)	-6.2 (0.87)	0.3 (1.00)	-5.1 (0.81)	8.4 (0.58)
Neuro-ophthalmology	4.4 (0.99)	-0.7 (1.00)	2.2 (1.00)	1.2 (1.00)	-9.1 (0.76)	10.0 (0.63)	-6.7 (0.75)	1.3 (1.00)
Oculoplastic	3.7 (0.81)	-1.4 (1.00)	3.2 (0.80)	-3.0 (0.87)	11.7 (0.34)	-2.5 (0.94)	2.0 (0.94)	13.7 (0.18)
Oncology	-11.5 (0.60)	9.5 (0.68)	-16.0 (0.38)	18.0 (0.44)	-1.9 (1.00)	-3.3 (1.00)	-2.4 (1.00)	-7.6 (0.78)
Retina	2.4 (0.80)	-6.5 (0.35)	7.9 (0.21)	-3.2 (0.69)	7.3 (0.38)	0.4 (1.00)	-0.9 (1.00)	7.4 (0.27)
Glaucoma	1.1 (1.00)	4.2 (0.62)	2.7 (0.78)	-1.9 (0.89)	3.9 (0.70)	-6.5 (0.46)	4.5 (0.62)	8.0 (0.32)

## DISCUSSION

The medical profession in Brazil is experiencing a notable demographic shift, especially with regard to the increasing feminization of medical specialties^(1)^. This study demonstrates that in ophthalmology, there has been not only a rise in the number of certified female ophthalmologists but also an increase in women holding key leadership and scientific roles. Recently, more female ophthalmologists have taken chair positions within regional and national societies, as well as ophthalmology subspecialty societies. Notably, for the first time in history, a woman was elected president of the CBO in 2024^(5)^.

As anticipated, this study identified a significant annual increase of 1.33% in female representation at the CBO from 2016 to 2023. This indicates that Brazil follows the global trend toward the feminization of ophthalmology, although at a slower pace compared to data from the AAO annual meeting, where female representation in all conference activities increased by 6.6% (p<0.001) between 2018 and 2021^(3)^.

Our results show a general increase in female participation at the Brazilian ophthalmology meeting and a narrowing of the gender gap. Moreover, the percentage of women participating in the 2023 meeting (42%) closely matched the proportion of female ophthalmologists practicing in Brazil that year (43%), demonstrating alignment between the national female ophthalmologist population and their representation at the country’s largest ophthalmology event. While these findings suggest that the rise in female representation at scientific meetings in Brazil is occurring naturally, we propose that political and strategic initiatives may have contributed to this growth. One such effort is the creation of the Brazilian Women in Ophthalmology Committee (“CBO Mulher”), established around 2014–2015 by a group of female ophthalmologists and supported by the CBO to advocate for women in the field^(6)^.

A recent study on female participation in ophthalmology in the United States reported that although women comprise only 27% of ophthalmologists in the United States, they represented about 38.7% of attendees at the AAO congress^(2^,^4)^. In comparison, the current study found that the proportion of female participation relative to practicing female ophthalmologists was lower in Brazil. It is important to consider, however that the AAO annual meeting has an international focus, which may make it less representative of the US medical population alone, whereas the CBO centers specifically on Brazilian physicians.

Given Brazil’s vast size, significant social and economic inequalities exist across its regions^(7)^. This study identified disparities in female representation when examining geographic distribution. Of the female participants in the CBO scientific program from 2016 to 2023, the majority were from the Southeast region (64%), followed by the Northeast (16%). This pattern reflects the overall distribution of ophthalmology residency programs in Brazil, with nearly half located in the Southeast and fewer than 20% in the Northeast. Similarly, the geo graphic distribution of ophthalmologists shows 52.1% practicing in the Southeast and 19.1% in the Northeast^(1)^. These results suggest that regional infrastructure and training availability may affect professional representation at national congresses.

The roles participants hold at meetings are important because they reflect hierarchy and seniority among ophthalmologists within scientific gatherings^(8)^. In this study, the speaker role showed significant growth, with an annual increase of 1.5 times per year. Notably, the coordinator role demonstrated the most substantial growth, rising from 22% female representation in 2016 to 40% in 2023. This role is highly regarded and prestigious, typically occupied by more experienced pro fessionals, which indicates positive advancement in the feminization of this important position^(8^,^9)^. In contrast, the moderator/discussant role experienced a smaller increase, with an annual growth rate of 1.24%, which was not statistically significant.

Considering that ophthalmology includes several clinical and surgical subspecialties with different characteristics, it is essential to analyze the proportion of women in these areas. The study found no statistically significant change in female participation across most subspecialties. This contrasts with international data, which generally show lower female involvement in subspecialties with higher surgical demands, such as retina and vitreous, and greater female presence in fields like pediatric ophthalmology. According to 2018 data from the American Board of Ophthalmology, only 20% of retina and vitreous specialists were female, compared to 46.2% of Pediatric Ophthalmologists^(3^,^8)^.

A limitation of this study is that it examined female ophthalmologist representation in only one ophthalmology meeting in Brazil; therefore, the findings cannot be generalized. However, this national ophthalmology meeting is the largest in the country, attracting approximately 6,000 participants annually(10). Another limitation was the inability to identify and exclude international participants, meaning some international attendees may have been included in the analysis. It is also important to note that for gender analysis, only male and female categories were considered, since other gender options were not available during registration for the CBO meetings. We recognize that the gender spectrum, including trans women, was not addressed, which represents another limitation of this study.

In conclusion, the analysis of female participation at the CBO from 2016 to 2023 shows a marked increase in women’s presence at this scientific event. Female representation has risen over the years not only overall but also in key roles such as Coordinators and Speakers, demonstrating tangible progress toward gender equality in Brazilian ophthalmology. In addition, there is a clear concentration of female ophthalmologists in the Southeast and Northeast regions of Brazil, which corresponds to the distribution of specialists and residency programs nationwide.

However, to attain effective gender representation in ophthalmology, it is crucial to implement strategies that encourage female participation across all areas of the specialty, especially in subspecialties that have traditionally been male-dominated. These initiatives will help create a fairer and more inclusive environment, supporting the professional and scientific growth of female ophthalmologists in Brazil.
